# Real-Time Estimation of Satellite-Derived PM_2.5_ Based on a Semi-Physical Geographically Weighted Regression Model

**DOI:** 10.3390/ijerph13100974

**Published:** 2016-09-30

**Authors:** Tianhao Zhang, Gang Liu, Zhongmin Zhu, Wei Gong, Yuxi Ji, Yusi Huang

**Affiliations:** 1 State Key Laboratory of Information Engineering in Surveying, Mapping and Remote Sensing, Wuhan University, Wuhan 430079, China; tianhaozhang@whu.edu.cn (T.Z.); weigong@whu.edu.cn (W.G.); jiyuxi_ss@163.com (Y.J.); mavis_huang@whu.edu.cn (Y.H.); 2 Shanghai Institute of Satellite Engineering, Shanghai 201100, China; liugang8243@126.com; 3 College of Information Science and Engineering, Wuchang Shouyi University, Wuhan 430064, China; 4 Collaborative Innovation Center for Geospatial Technology, Wuhan 430079, China

**Keywords:** real-time estimation, national-scale PM_2.5_, aerosol optical depth, fusion by DT and DB, semi-physical geographically weighted regression

## Abstract

The real-time estimation of ambient particulate matter with diameter no greater than 2.5 μm (PM_2.5_) is currently quite limited in China. A semi-physical geographically weighted regression (GWR) model was adopted to estimate PM_2.5_ mass concentrations at national scale using the Aqua Moderate Resolution Imaging Spectroradiometer (MODIS) Aerosol Optical Depth product fused by the Dark Target (DT) and Deep Blue (DB) algorithms, combined with meteorological parameters. The fitting results could explain over 80% of the variability in the corresponding PM_2.5_ mass concentrations, and the estimation tends to overestimate when measurement is low and tends to underestimate when measurement is high. Based on World Health Organization standards, results indicate that most regions in China suffered severe PM_2.5_ pollution during winter. Seasonal average mass concentrations of PM_2.5_ predicted by the model indicate that residential regions, namely Jing-Jin-Ji Region and Central China, were faced with challenge from fine particles. Moreover, estimation deviation caused primarily by the spatially uneven distribution of monitoring sites and the changes of elevation in a relatively small region has been discussed. In summary, real-time PM_2.5_ was estimated effectively by the satellite-based semi-physical GWR model, and the results could provide reasonable references for assessing health impacts and offer guidance on air quality management in China.

## 1. Introduction

Many researches have proven that atmospheric particles from both anthropogenic emissions and natural sources are tightly linked to environment deterioration and climate change [1,2,3]. Some epidemiological studies have shown that fine particulate matter with an aerodynamic diameter no greater than 2.5 μm (PM_2.5_) has a positive correlation with respiratory and cardiovascular disease occurrence [4,5,6]. The measurement of ground-level PM_2.5_ mass concentrations is therefore of vital significance for effective air quality management. With a rapidly developing economy, China has been experiencing severe PM_2.5_ pollution that has aroused widespread public concern [7]. The quality of PM_2.5_ estimation often decreases along with the extent of spatial distance, although stationary ground measurements are reasonably accurate [8]. Consequently, acquiring an accurate estimation of PM_2.5_ exposure characterization at the national scale for China is necessary [9].

Since remote sensing from a satellite could generally compensate for the limited spatial coverage by ground-level monitoring, this efficient method was also adopted to estimate PM_2.5_ mass concentrations [10,11,12,13]. Since satellite aerosol optical depth (AOD) measures the light extinction of a specific atmospheric column, it has a direct relation with the concentrations of particulate matter in that column, and further to the mass concentration of particles [10,11]. In addition, to adopt empirical models to estimate ground PM_2.5_ based on satellite-derived AOD [14,15,16], many meteorological and geographical parameters have been incorporated to develop superior models for better estimations of PM_2.5_ mass concentrations, such as the generalized additive model (GAM) [17], mixed effects model (MEM) [18], and artificial neural network model (ANN) [14,19]. Since the relationship between AOD and PM_2.5_ is not spatially constant [8], the geographically weighted regression (GWR) model, which better constrains the spatial variability in a large-region regression, has been adopted to estimate geographical elements in large regions [20,21]. In addition, the hygroscopic effect of the aerosol extinction coefficient for particulate matter has been corrected by relative humidity, and the particulate matter vertical distribution has been taken into consideration from a physics perspective [22]. Furthermore, a nation scale research on the GWR model combined with physical corrections indicated that such corrections by atmospheric vertical feature and relative humidity (RH) could be effectively embedded in the GWR model for better performance [23]. Nevertheless, almost all previous studies adopted the datasets with daily, monthly, or even seasonal average values, which ignores the real-time effect of meteorological parameters. To the best of our knowledge, real-time PM_2.5_ should exactly correspond to satellite-derived AOD when the satellite overpasses a ground-level monitoring station.

In this paper, we first fused Aqua AOD products based on the Dark Target (DT) and Deep Blue (DB) algorithms, and aligned them to real-time PM_2.5_ based on five time zones. The spatial distribution of PM_2.5_ was then estimated by the semi-physical GWR model combined with reanalysis of meteorological parameters. For quantitatively evaluating the model performance, a 10-fold cross-validation was adopted to demonstrate the relationship between measured PM_2.5_ and estimated PM_2.5_ in four seasons. Moreover, the seasonal spatial distribution of the satellite-derived PM_2.5_ at the national-scale was analyzed and discussed, as well as the comparisons with ground measurements from the perspective of model mechanism.

## 2. Materials and Methods

### 2.1. Data Collection and Processing

#### 2.1.1. Hourly Ground-Level PM_2.5_ Measurements

**Figure 1:**
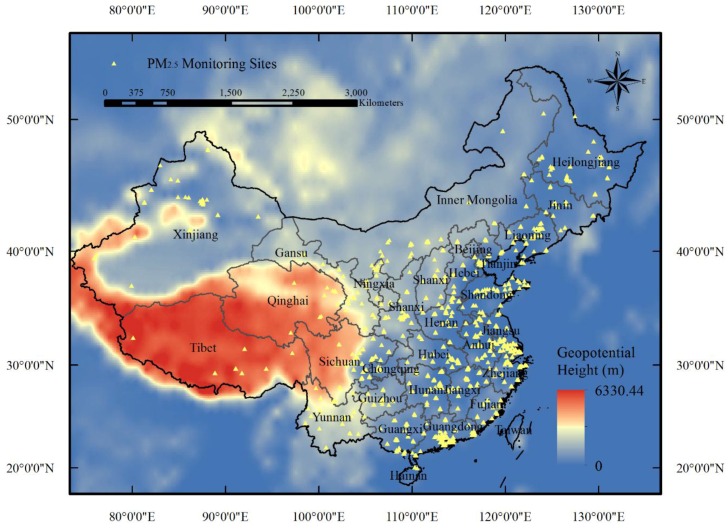
Spatial distribution of 1344 monitoring sites (solid yellow triangles) of ambient particulate matter with diameter no greater than 2.5 μm (PM_2.5_) utilized in this study.

A monitoring network of national-scale air quality has been established, and a report of the hourly mass concentrations of PM_2.5_ since 2013 has been officially released to the public by the Chinese Ministry of Environmental Protection (MEP). As shown in Figure 1, more than 1300 ground monitoring stations were set up to cover residential areas by the end of 2014, and monitoring data measured by the tapered element oscillating microbalance method (TEOM) or the beta-attenuation method are accessible in the China Environmental Monitoring Center (CEMC, Chaoyang district, Beijing) [24].

#### 2.1.2. Satellite-Derived AOD

Since the Moderate Resolution Imaging Spectroradiometer (MODIS) has been shown to provide aerosol products with assured quality compared with other satellite sensors [25,26,27], the AOD derived from MODIS was chosen for this study. The Collection 6 (C6) datasets from MODIS were released last year, and the aerosol products, including AOD, have been generally shown to reach the required accuracy through the validation against the ground monitoring observations of sun photometers from the Aerosol Robotic Network (AERONET) in China [28,29]. The DB and DT algorithms have been adopted for the C6 MODIS reprocessing, and the second generation DB was expanded to cover brighter desert/urban areas and vegetated land surfaces, which preferably makes up for deficiencies of the DT algorithms [30,31]. In addition, a fusion dataset combining these two algorithms, which unfortunately provides AOD at a 10 km spatial resolution, is also included in the C6 product. Throughout this study, the Aqua AOD datasets were distributed in Hierarchical Data Format (HDF) format from the NASA LAADS website [32] on the national scale (longitude (73°40′ E–135°2.5′ E), latitude (3°52′ N–53°33′ N)). As demonstrated in Table 1, there are four science data segments (SDS) utilized for AOD fusion, where “3k” and “10k” represent the Level 2 aerosol products with 3 km and 10 km spatial resolutions, respectively. Since AOD uses the DB algorithm, and AOD generated from the DB and DT algorithms both only possess a 10 km spatial resolution, this study set out to combine the 3 km DT AOD with the 10 km DB AOD using the concept of complementary advantages. To ensure the accuracy of AOD, only those retrievals that reach required quality assurance (QA) were used (corresponding to flag QA = 3 for DT; flag QA = 2 or QA = 3 for DB) [33,34].

#### 2.1.3. Meteorological Factors

The precipitation dataset used in this study was obtained from a daily averaged precipitation grid dataset. The dataset, which is released by the National Meteorological Information Center [35], is spatially interpolated by the Thin Plate Spline (TPS) method using data from 2472 ground-level meteorological stations in China with 0.5° spatial resolution. Other meteorological data, consisting of surface relative humidity (RH_Surface), u- and v-components of surface winds, surface temperature, planetary boundary layer height (PBLH), and atmospheric pressure, were collected from National Centers for Environmental Prediction (NCEP) reanalysis datasets. Atmospheric product, oceanic, and land surface on every 6 hours with spatial resolution of 1.0° is available in the NCEP dataset, which is accessible on their website [36].

#### 2.1.4. Data Integration

Since Aqua MODIS passes the equator at approximately 1:30 p.m. local time, this study correspondingly selected an averaged measured PM_2.5_ values at 1:00 p.m. and 2:00 p.m. local time. It should not be ignored that the whole area of China stretches across five time zones, whereas the measurements of PM_2.5_ are recorded in Beijing time (UTC + 8 h). Therefore, it is necessary to adjust measuring time to local time. Additionally, since the chosen datasets have different spatial resolutions, the bilinear interpolation was adopted to ensure spatial consistency of meteorological parameters. Moreover, the meteorological parameters and AOD values were both selected from the pixel in the grid where the ground-level monitoring stations geographically locate.

### 2.2. Methodology

#### 2.2.1. Physics-Based Corrections

According to previous studies, physics corrections based on vertical distribution and relative humidity could make remarkable improvements in the quality of datasets and model performance, especially the relationship between AOD and PM [23,37]. Since the ground level environmental monitoring station has measured the PM_2.5_, it is the mass concentration data of near ground PM_2.5_ that has been collected. Therefore, optical parameters for the ambient atmosphere should be measured instead of satellite-derived AOD which characterizes the entire atmospheric column. Thus, a vertical revision formula was expressed as follows to correct the satellite-derived AOD [11]:

(1) $$\mathrm{Revised}\_\mathrm{AOD}\;=\;\frac{AOD}{PBLH}$$

The dry PM_2.5_, measured by the TEOM method, is set as the standard in Chinese air-quality stations and ignores the hygroscopic effect of particulates. A relative RH revised PM_2.5_ can be calculated by a general revision formula [38]:

(2) $$\mathrm{Revised}\_PM_{2.5}=PM_{2.5}\times {\left(1-\frac{RH}{100}\right)}^{-1}$$

#### 2.2.2. Model Structure and Validation

A real-time GWR model has been established in this study considering the physical revision above, and estimated coefficients of a continuous surface and generate a local R^2^ could be calculated by weighing the contribution of every observation. The structure of the GWR model developed in this study can be expressed in the following equation:

(3) $$\begin{array}{cc} Revise\_{\mathrm{PM}}_{2.5,l,d} & =\beta_{0,l,d}+\beta_{1,l,d}Revise\_AOD_{l,d}+\beta_{2,l,d}Last\_Prec_{l,d}\\ & \beta_{3,l,d}ST_{l,d}+\beta_{4,l,d}PS_{l,d}+\beta_{5,l,d}WS_{l,d} \end{array}$$

where the $Revise\_{\mathrm{PM}}_{2.5,l,d}$ (μg/m^3^) is the ground-level PM_2.5_ concentration revised by RH at location *l* on day *d* at 1:30 p.m. local time; $\beta_{0,l,d}$ denotes the intercept at location *l* on day *d* at 1:30 p.m. local time; $\beta_{1,l,d}$ to $\beta_{5,l,d}$ represent location-specific slopes; $Revise\_AOD_{l,d}$ (no unit) is the Aqua-MODIS AOD fused products revised by PBLH (km) at location *l* on day *d* at 1:30 p.m. local time; $Last\_Prec_{l,d}$ is the daily total precipitation at location *l* on the day before day *d*; and $ST_{l,d}$, $PS_{l,d}$, and $WS_{l,d}$ are the surface temperature (K), atmospheric pressure (Pa), and surface wind speed (m/s), respectively, at location *l* on day *d* at 1:30 p.m. local time.

Additionally, a 10-fold cross-validation method [39] has been employed to validate the quality of the model by comparing the estimated PM_2.5_ against the monitoring values. The dataset was divided into 10 folds in four seasons, and the model was fitted by nine folds with one fold set for validation in each cross-validation circle. This process was completely repeated 10 times, when every fold was validated. Furthermore, the estimation equation, decision coefficient R^2^, and mean absolute error (MAE, μg/m^3^) were calculated to evaluate the model performance.

## 3. Results and Discussion

### 3.1. Descriptive Statistics

**Figure 2:**
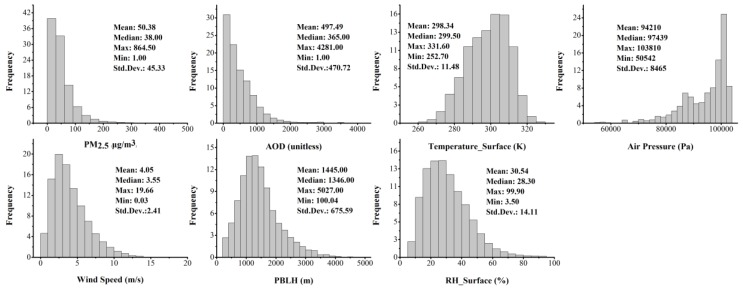
Histograms and descriptive statistics for PM_2.5_, AOD, surface temperature, atmospheric pressure, wind speed, planetary boundary layer height (PBLH), and surface relative humidity (RH) in the model fitting.

Histograms and descriptive statistics for the variables used in the GWR model are displayed in Figure 2, except for the precipitation dataset, since most of the precipitation for the previous day equaled to zero. Overall, the mass concentrations of PM_2.5_ ranged from 1 to 864.5 μg/m^3^ with an annual average of 50.38 μg/m^3^ and standard deviation (SD) of 45.33 μg/m^3^. The AOD frequency histograms have a similar shape as measured PM_2.5_, with an annual average AOD value of 497.49 and SD of 470.72. The annual atmospheric pressure displays a bimodal distribution for the whole part of China, which is possibly caused by the large elevation difference in Sichuan, Qinghai, and Tibet (Figure 1). The other meteorological variables, including surface temperature, wind speed, PBLH, and surface RH, were approximately log-normal or Weibull distributed.

### 3.2. Model Fitting and Validation

**Figure 3:**
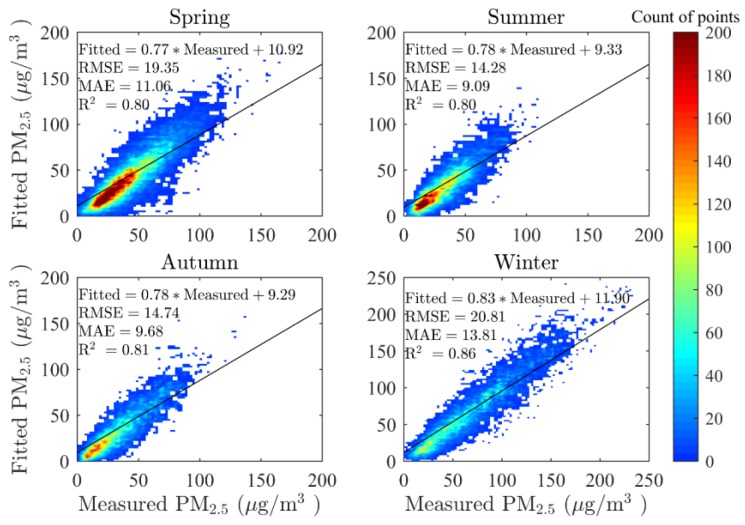
Scatter plot of cross-validation results in the real-time geographically weighted regression (GWR) model in four seasons. MAE: mean absolute error (μg/m^3^); RMSE: root mean square error (μg/m^3^). The solid line indicates the linear regression results.

Figure 3 shows the cross-validation (CV) results for the real-time GWR model in four seasons. In this study, the four seasons were defined based on both astronomy and climate method conjunctively, namely, spring (March, April, and May), summer (June, July, and August), autumn (September, October, and November) and winter (December, January, and February). According to the model fitting, the CV R^2^ values are 0.80, 0.80, 0.81, and 0.86 for spring, summer, winter, and autumn, respectively, and the CV RMSE values are 19.35, 14.28, 14.74, and 20.81 μg/m^3^ for the four seasons, respectively. The fitted results in this study could therefore account for over 80% of the variability in the corresponding PM_2.5_ mass concentrations. These fitting results were relatively good at the national scale when compared to other studies in China (cross validation R^2^ achieved 0.64 and 0.79 in annual average, respectively) [24,29]. Generally speaking, summer possessed the lowest mass concentration of PM_2.5_, followed by autumn. PM_2.5_ concentrations were higher in both spring and winter, however, most values in spring remained low. The decision coefficient, R^2^, was a little higher in winter than in other seasons, which was possibly caused by fewer satisfactory points in the northeastern and western regions of China, because MODIS could hardly retrieve AOD on surfaces with strong reflectance, such as snow-covered surfaces that are predominant in those regions during winter time. However, the maximum CV MAE value was reached in winter while the lowest value of MAE appeared in summer (9.09 μg/m^3^). It is apparent that the CV MAE had no relationship with CV R^2^, but was linked to the value of PM_2.5_. In other words, the CV MAE demonstrated a proportional relation to the mass concentration of PM_2.5_, rather than to model performance.

It should not be ignored that the slopes of the linear fitting equation are all near 0.8 and the intercepts range from 9 to 12 μg/m^3^, which indicates no apparent differences in the seasonal model-fitting results. Similar to many CV equations determined in previous studies using GWR models [29,37,40], the slope is less than 0.9 while intercept is positive. In this study, when the monitored PM_2.5_ was less than 50 μg/m^3^, our model tended to overestimate, but to underestimate when the monitored PM_2.5_ was larger than 50 μg/m^3^ on general situations. This phenomenon could possibly be explained by the mechanism of GWR model that variable coefficients tending to be similar when they are geographically close to each other. Therefore, estimated values would be more even when measurements differ sharply from each other in a small region. In other words, the overall estimated PM_2.5_ would appear to be slightly averaged from the measured PM_2.5_.

### 3.3. Seasonal Estimation of PM_2.5_ Mass Concentrations

**Figure 4:**
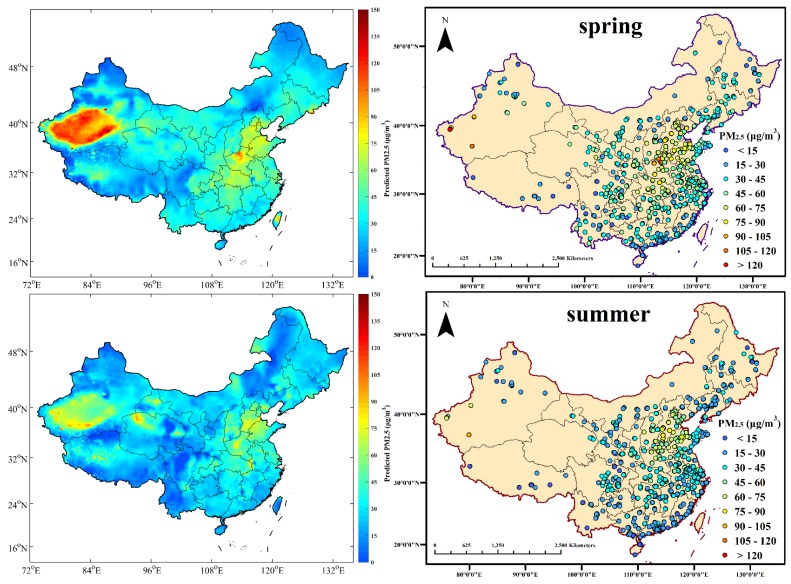

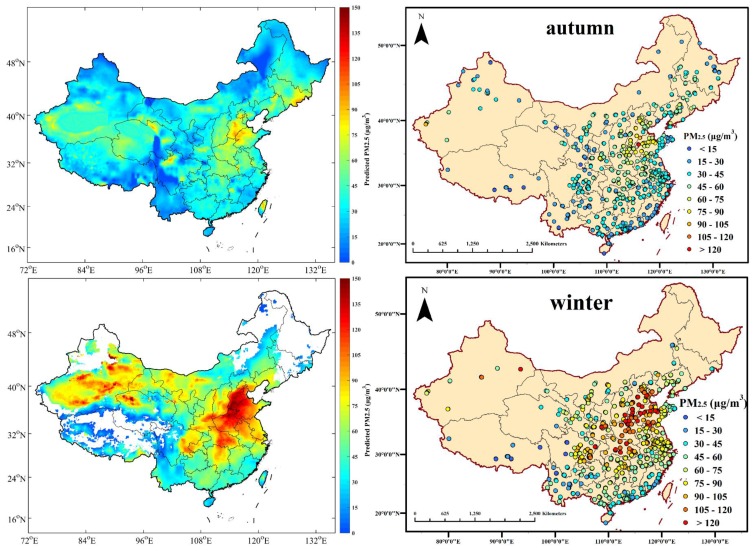
Comparison of seasonal average AOD-derived PM_2.5_ and ground-measured PM_2.5_ concentrations from real-time datasets, when the corresponding AOD values are available.

The estimated seasonal average PM_2.5_ concentrations are illustrated in Figure 4. In order to evaluate the accuracy in the estimation of the GWR model, the corresponding ground-level PM_2.5_ measurements are also shown in Figure 4 as a comparison standard. The spatial and temporal variations of PM_2.5_ derived from Aqua MODIS AOD products are generally quite similar to those from the ground-level measurements. According to the World Health Organization (WHO) Air Quality Interim Target levels (IT), WHO IT-1 and WHO IT-3 set the PM_2.5_ standards of 35 μg/m^3^ and 15 μg/m^3^, respectively [41]. In winter, China suffers severe environmental pollution by fine particles, with related values in most regions maintaining over 60 μg/m^3^, exceeding the recommended standards by approximately 58% or 300%, respectively. A major source of PM_2.5_ in winter was probably winter heating, while the winter meteorological conditions impeded the diffusion of atmospheric pollution to a certain extent [42,43,44,45,46]. The highest values of PM_2.5_ arose in the Xinjiang Autonomous Region, followed by the Jing-Jin-Ji Region (including Beijing, Tianjin, and Hebei) and Central China (including Hunan, Hubei, and Henan). The Tarim Basin, which is located in the southern section of the Xinjiang Autonomous Region, is covered mostly by the Taklimakan Desert. The seasonal mean PM_2.5_ in the Tarim Basin was on average greater than 60 μg/m^3^ in summer and autumn, and greater than 85 μg/m^3^ in spring and winter. The Taklimakan Desert, which occupies over 60% of the Basin and spans the Tarim Basin, Gansu province, Ningxia province, and western Inner Mongolia, additionally influences the pollution levels with dust storms in spring and winter. Dust aerosols come mostly from primal generation and entrained effects across eastern Asia [47], and contribute greatly to PM_2.5_ levels in Northern China. In the Jing-Jin-Ji Region, the seasonal average PM_2.5_ mass concentrations are generally higher than 100 µg/m^3^ in winter and higher than 75 µg/m^3^ in other seasons. High levels of industrialization and urbanization, as well as high density of human activities, have led to severe PM_2.5_ pollution in these areas [48,49]. The cleanest regions lie in Heilongjiang, Yunnan, Tibet, and Hainan, where the seasonal average PM_2.5_ values are generally lower than 35 µg/m^3^.

Although the model has been proven to estimate spatial distribution of PM_2.5_ effectively in China, there still existed prediction errors in several regions. For instance, the AOD-derived PM_2.5_ displayed fleck effects in Sichuan, Qinghai, and Gansu, indicating over fit in these provinces. In other words, there were often maximum or minimum values emerging among the ordinary values. This phenomenon in model prediction could be explained by the two possible reasons as follows. First, ground level PM_2.5_ monitoring sites are distributed unevenly, with primary coverage in large urban centers and sparse coverage in rural areas, especially in the western regions of the country including western Sichuan, Tibet, and Qinghai. Since the GWR model adopts an adaptive bandwidth searching method, the bandwidth will increase when the ground stations are sparse, further decreasing the effects of model performance. However, this problem could be gradually resolved by the construction of a Chinese air-quality monitoring network [50]. In current data conditions, the model could only achieve anticipative efficacy of estimation in eastern China. Secondly, since climatic conditions change with changes in elevation, sharp elevation changes in a relatively small region could affect the model’s independent variables, especially meteorological parameters. This issue would ultimately lead to an irresolvable situation of bandwidth selecting in the GWR model [23]. Moreover, although the DT AOD retrieval algorithm could lead to lack of estimation over bright surfaces such as the Taklimakan Desert, the fused AOD in this study displayed good estimation of PM_2.5_ in desert regions all year round. Furthermore, since the coverage of satellite-derived AOD is restrained by surface reflectance conditions, it is the sampling limitation according to the retrieval algorithm that causes missing estimation values for PM_2.5_ in plateaus and parts of the northern regions in winter [51].

## 4. Conclusions

A real-time estimation of mass concentrations of PM_2.5_ using physical corrections at the national scale, combined with fused Aqua MODIS AOD and meteorological parameters, has been conducted by GWR model. Over 80% of the variability in the corresponding PM_2.5_ mass concentrations at 1:30 p.m. local time could be explained by the real-time GWR model. The MAE, via a 10-fold cross-validation, demonstrated a proportional relation to the mass concentrations of PM_2.5_, instead of to the model performance in each season. Moreover, the model fitting did not appear to be influenced by seasons, with the slopes of the linear fitting equations remaining near 0.8 while the intercepts were in a tight range, from 9 to 12 μg/m^3^. These fitting results indicate that GWR also tended to overestimate for low measurements and underestimate for high measurements. In other words, the overall estimated PM_2.5_ would appear to be slightly averaged from the ground measured PM_2.5_.

According to the spatial distributions of seasonal average PM_2.5_ mass concentrations for the real-time GWR model, most regions in China had a concentration of over 60 μg/m^3^ in winter, which exceeds the recommended standards of the WHO, which was probably caused by winter heating and meteorological conditions. The highest values of PM_2.5_ appeared in the Xinjiang Autonomous Region, followed by the Jing-Jin-Ji Region and Central China. The cleanest regions were in Tibet, Heilongjiang, Yunnan, and Hainan, where the seasonal average PM_2.5_ values were generally lower than 35 µg/m^3^. Moreover, the spatial distributions of seasonal average mass concentrations of PM_2.5_ for the real-time GWR model indicate a fleck effect, namely over-fitting phenomenon, in the provinces of Sichuan, Gansu, and Qinghai. This could be prevailingly caused by the spatially uneven distribution of PM_2.5_ monitoring sites and sharp elevation changes in a relatively small region. Thus, in current data conditions, it is hard to achieve anticipative performance of estimation in western China. Furthermore, the estimation of PM_2.5_ by fusing AOD from both DT and DB algorithms could provide better coverage in the desert regions such as the Taklimakan Deserts, nevertheless, it still has limitations on plateaus and parts of the northern regions in winter due to restraints from surface reflectance conditions.

In summary, real-time PM_2.5_ was effectively estimated in national scale by the developed semi-physical GWR model using the fused Aqua MODIS AOD product combined with NCEP reanalysis meteorological parameters. In order to achieve better performance, further research on the modeling algorithm, as well as a source analysis of PM_2.5_, are currently in progress. The real-time estimation of PM_2.5_ mass concentrations at the national scale could provide a reasonable reference for health impact assessment, air quality management and emission control strategies in China.

## Figures and Tables

**Table 1:** Level 2 science data segments (SDS) titles and explanations for Aqua Moderate Resolution Imaging Spectroradiometer (MODIS) aerosol optical depth (AOD).

SDS Title	Explanations
Image_Optical_Depth_Land_And_Ocean (3k)	AOD at 550 nm for both ocean and land with all quality data using the DT algorithm.
Land_Ocean_Quality_Flag (3k)	Quality flag for land and ocean aerosol retrievals (0 = bad, 1 = marginal, 2 = good, 3 = very good)
Deep_Blue_Aerosol_Optical_Depth_550_Land (10k)	AOD at 550 nm for land with all quality data using the DB algorithm
Deep_Blue_Aerosol_Optical_Depth_550_Land_QA_Flag (10k)	Deep Blue aerosol confidence flag (0 = no confidence, 1 = marginal, 2 = good, 3 = very good)
